# Unipolar resistive switching of ZnO-single-wire memristors

**DOI:** 10.1186/1556-276X-9-381

**Published:** 2014-08-07

**Authors:** Yong Huang, Ying Luo, Zihan Shen, Guoliang Yuan, Haibo Zeng

**Affiliations:** 1 Institute of Optoelectronics & Nanomaterials (ION), School of Materials Science and Engineering, Nanjing University of Science and Technology, Nanjing 210094, China; 2 Department of Fundamental Courses, Jinling Institute of Technology, Nanjing 211169, China

**Keywords:** ZnO, Resistive random access memory (RRAM), Resistive switching (RS), Electrical properties

## Abstract

Well unipolar resistive switching (RS) behaviors were observed from Ag/ZnO single-microwire/Ag memristors. The reset voltages were larger than the set voltages, and all of them were less than 1 V. The resistance ratios of high-resistance state (HRS) to low-resistance state (LRS) reached 10^3^. The bistable RS behaviors were entirely reversible and steady within 100 cycles. It was found that the dominant conduction mechanisms in LRS and HRS were ohmic behavior and space-charge-limited current (SCLC), respectively.

## Background

Resistive random access memory (RRAM) and memristor have attracted rapidly increasing attention due to their high-speed operation, high-density storage, and low-voltage driving virtues for nonvolatile memory (NVM) applications [1,2]. Generally, a memristor is composed of a metal-insulator-metal (MIM) cell, where the NVM effect comes from their ability of reversible resistive switching (RS) between low-resistance state (LRS or R_ON_) and high-resistance state (HRS or R_OFF_) under voltage stimulus. Among the various candidate materials for RRAM and memristor, zinc oxide (ZnO) has promising advantages, such as facile synthesis, reversible and steady RS property, and low set and reset voltages [3-5]. Up to now, memristors based on ZnO thin films have been reported according to their RS behaviors from intrinsic defects (e.g., oxygen vacancies) and extrinsic impurities (e.g., Ag^+^ ions) [6-8]. However, several serious problems for memristors still exist. First of all, the RS mechanisms are still subjects of heated debate. Second, the operating voltages are usually too large and expected to be less than 1 V. Finally, the RS behavior in a single ZnO microwire has seldom been reported, but could have special applications due to its one-dimensional structure which include memristors, nanolasers, photodiodes, nanogenerators, gas sensors, acoustic resonators, piezoelectric gated diodes, etc. [5,9].

In this paper, we report on a ZnO single-wire memristor with low driving voltage and high stability as well as its interesting RS behaviors. Well unipolar RS properties were observed, including the set and reset voltages less than 1 V, resistance ratio as high as 10^3^, and strong endurance stability within 100 cycles. Abnormally, the reset voltages are observed to be larger than the set voltages, which are contrary to most previous reports and are explained by the space-charge-limited current (SCLC).

## Methods

**Figure 1:**
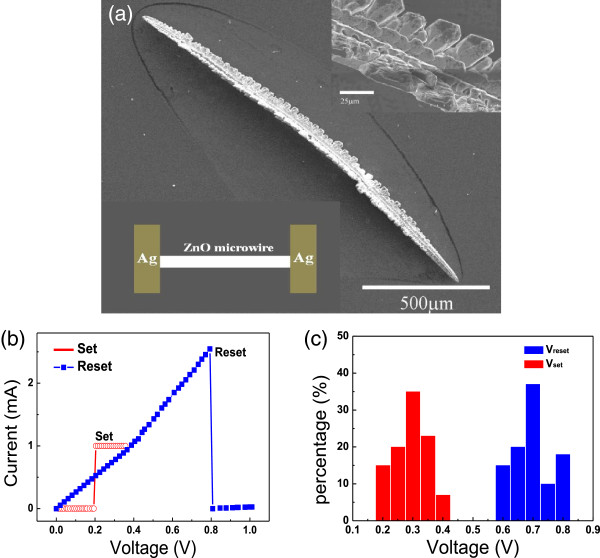
**SEM image and unipolar RS behaviors of ZnO microwire and distribution of set and reset voltages. (a)** SEM image of an individual ZnO microwire. The upper and lower insets show the magnified image of the microwire and schematic diagram of Ag/ZnO/Ag device, respectively. **(b)***I*-*V* characteristics of the Ag/ZnO/Ag memristor. **(c)** The distribution of the set and reset voltages.

ZnO microwires were synthesized in a horizontal quartz tube furnace (6 cm in diameter and 60 cm in length) by a vapor-phase transport method as reported elsewhere [5,10]. An individual ZnO microwire was put on a glass substrate. Two drops of silver paste were coated on the two ends with a spacing of about 1 mm. After being baked at 120°C for 10 min, the silver paste became solid, forming the memristor devices as presented by the schematic diagram in the lower inset of Figure 1a. The material and device morphology was examined by scanning electron microscopy (SEM). The current-voltage (*I*-*V*) and endurance characteristics of the device were measured by a Keithley 2635 source meter (Keithley Instruments, Inc., Cleveland, OH, USA) and a probe station at room temperature in a voltage sweep mode. Each voltage sweep (50 points, 100 ms/point) began from 0 V, and the bias (1 V) was applied to one of the Ag electrode while the other was grounded. The maximum current was limited by a compliance current (CC) to avoid a permanent hard breakdown when unipolar HRS switched to LRS.

## Results and discussion

Figure 1a shows the SEM image of a typical ZnO microwire, whose length is about 1.5 mm and diameter is about 20 μm. Interestingly, as clearly confirmed by the upper inset of Figure 1a, hierarchical structures can be observed in the microwire. The formation of such ZnO hierarchical microwires can be attributed to the fast growth habit in <001 > direction and second nucleation on the side surfaces.

Figure 1b presents the typical unipolar RS behaviors of the device. First, electrical stress was loaded through a 1.5-V-forming voltage to induce an LRS. The current compliance was restricted at 1 mA to prevent permanent breakdown. Subsequently, in such an LRS, when the voltage was swept from zero to positive values (1 V), the leakage current increased approximately linearly and then very abruptly dropped approaching to zero at 0.8 V (reset voltage, *V*_reset_). Such an abrupt current drop indicated that the device had been switched into HRS, which is a nonvolatile off state and will be inherited in the early stage of the next voltage sweeping. Finally, during the second voltage sweep, a sudden current increase at about 0.2 V (set voltage, *V*_set_) appeared. Such a sudden increase over the compliance value demonstrated that the device was switched into LRS again, which is the nonvolatile on state and can also be memorized in the following cycle. Furthermore, when sweeping the voltage to negative voltages, similar RS behaviors, including on-off switching and state memorizing, were also observed.

Besides the above typical RS, some unusual phenomena were also observed. First, *V*_reset_ was found to be always larger than *V*_set_ as shown in Figure 1c, which is entirely different from the reported unipolar RS from MIM thin films [3]. Second, *V*_reset_ and *V*_set_ distribute in 0.62 to 0.8 V and 0.19 to 0.4 V, respectively. Both of them are less than 1 V, which will be very beneficial for the future application with low energy cost. Importantly, there is no overlap between these two ranges. Such obviously separated *V*_reset_ and *V*_set_ warrant a high reliability for device operation and, hence, also beneficial to application. Finally, the *V*_set_ distribution width is slightly larger than that of the *V*_reset_, which demonstrates that conducting filaments (CF) dominate the RS of such ZnO microwire memristors prepared in this study. According to the CF model [3,11,12], the formation of filaments (set) is more random than their rupture (reset) process due to the competition of different filamentary paths during the formation process.

**Figure 2:**
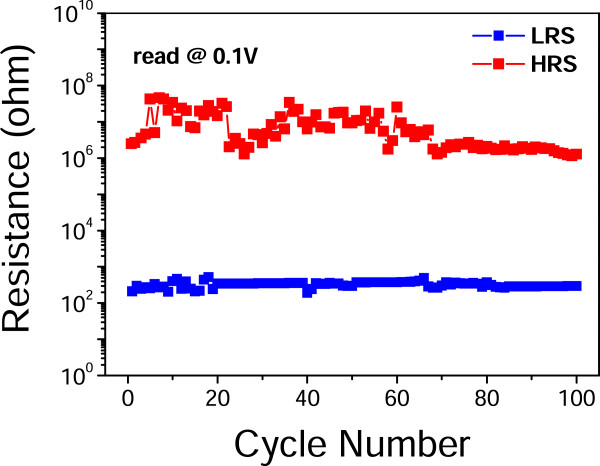
Resistances of LRS and HRS of Ag/ZnO/Ag device in 100 cycles.

These ZnO microwire memristors exhibited very high stability as shown in Figure 2. The on and off resistance values were read at 0.1 V in 100 DC sweeping cycles. The reading values of HRS appear to fluctuate from 1.2 to 28 MΩ in the early cycles, but become quite steady after 60 cycles, exhibiting very small fluctuations from 0.2 to 0.5 MΩ. When compared with previous reports [3], the LRS reading values here are relatively stable. Moreover, the on/off resistance ratios of HRS to LRS are as large as 10^3^ to 10^4^. Such high stability and large on/off ratios will greatly benefit the nonvolatile storage.

**Figure 3:**
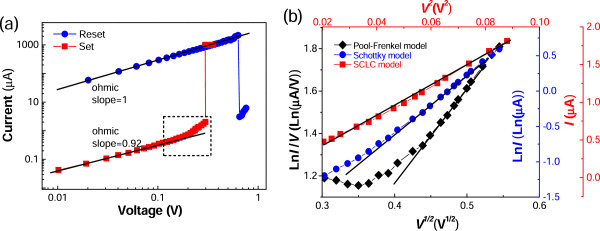
***I-V *curves in a log-log scale and *I-V *curves of HRS under a high-electric field. (a) ***I*-*V* characteristics of the Ag/ZnO/Ag device in log scale. **(b)** The plots of ln*I*-*V*^1/2^, ln*(I/V)*-*V*^1/2^, and *I*-*V*^2^ for the Schottky, PF, and SCLC conduction mechanisms, respectively.

To further understand the switching mechanisms, the *I-V* curves were re-plotted in a log-log scale as shown in Figure 3a. The low-voltage regions in both LRS and HRS can be well fitted linearly, and all slopes are close to 1. This implies that the conduction mechanisms of both LRS and HRS in the low-electric field region are ohmic behavior. Furthermore, the fitting line can run through the whole *I-V* curve of the LRS, indicating that ohmic behavior is still effective for the LRS under a high-electric field, which is consistent with the typical CF model [3,11,12]. Therefore, only the electron transport of HRS under a high-electric field, marked by a frame in Figure 3a, is abnormal and needs more explanation.

For such nonlinear *I-V* characteristic of HRS under a high-electric field, there are three leakage mechanisms, namely, space-charge-limited current (SCLC) [13], Schottky emission [14], and Poole-Frenkel (PF) emission [15]. The corresponding *I-V* curves can be described following different relations, where *e* is the electronic charge, ϵ_*r*_ is the relative dielectric constant, ϵ_0_ is the permittivity of free space, *d* is the film thickness, *k* is Boltzmann’s constant, and *T* is the temperature. Obviously, there are linear relationships of ln*I* vs *V*^1/2^, ln*(I/V)* vs *V*^1/2^, and *I* vs *V*^2^ for Schottky, PF, and SCLC mechanism, respectively.

(1) $$\left(\mathrm{Schottky}\right)\;\ln I\propto \frac{e\sqrt{\left(\mathrm{eV}\right)/\left(4\pi \epsilon_{r}\epsilon_{0}d\right)}}{\mathrm{kT}}$$

(2) $$\left(\mathrm{PF}\right)\;\ln I/V\propto \frac{e\sqrt{\left(\mathrm{eV}\right)/\left(\pi \epsilon_{r}\epsilon_{0}d\right)}}{\mathrm{kT}}$$

(3) $$\left(\mathrm{SCLC}\right)I\propto \epsilon_{r}\epsilon_{0}\mu V^{2}$$

**Figure 4:**
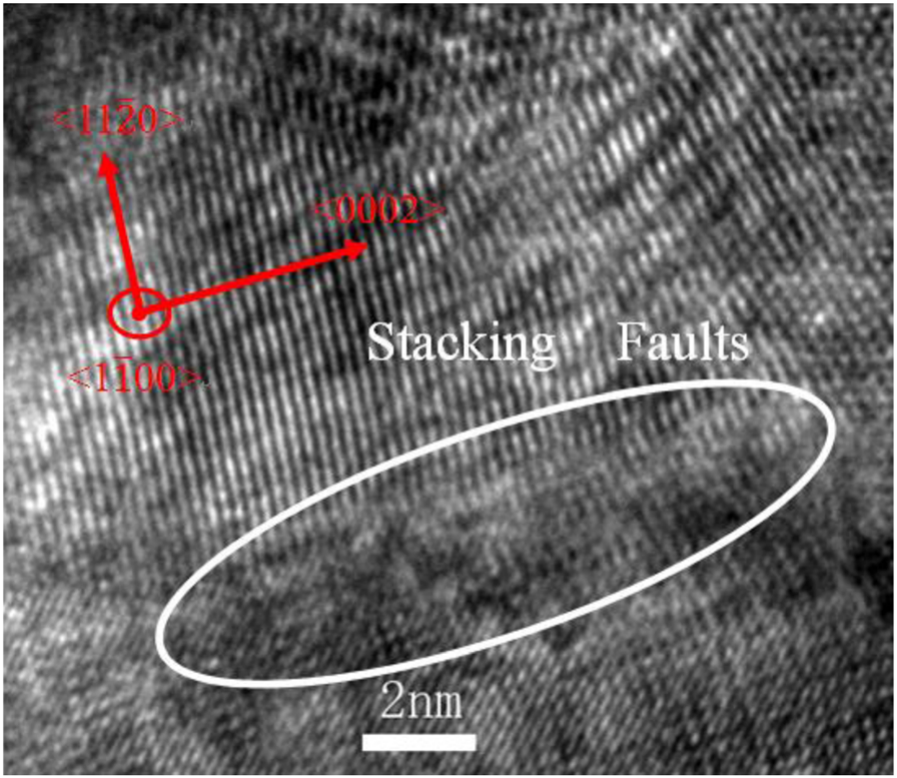
HRTEM image for a tiny part in the ZnO microwire.

The *I-V* curves of HRS under a high-electric field were re-plotted in these three kinds of scales as shown in Figure 3b. Very obviously, among these three re-plotted curves, the linearity degree of the *I* vs *V*^2^ curve is the highest, which demonstrates that the conduction mechanism of HRS in a high-electric field is dominated by SCLC mechanism.Figure 4 is the HRTEM image for a tiny part in the ZnO microwire. A number of crystal defects such as dislocations and stacking faults could be found in it. Even though a few stacking faults are terminated by partial dislocations, many of them are typically extended at about 10 nm between the two bounding partial dislocations. A plausible model for the occurrence of stacking faults is ascribed to condensation of vacancies or interstitials in the ZnO microwires thus leading to a missing or inducing additional (0002) plane. These extra stacking faults may provide good paths for electron transfer in the wires, and this fact results in a decrease of the electrical resistivity. Accordingly, the switching behaviors can be described as follows. The as-prepared ZnO microwire is insulating and contains many oxygen vacancy traps. Under the driving of a forming voltage, the abundant oxygen vacancies would be driven toward the cathode to assemble a conducting channel through the microwire’s grain boundaries, and hence, the device switches from the off to the on state. That is, the defects align to form tiny conducting filaments in the HRS and these tiny conducting filaments gather together to form stronger and more conducting filaments leading to the transition to the LRS. However, with the limit of compliance current, the loss of oxygen is not that serious that the HRS can be recovered through the redistribution of oxygen vacancies because of the passing of higher current and the Joule heating in the following voltage sweep, which corresponds to the reset process, whereas the so-called set process corresponds to the recovery of conductive filaments.

## Conclusions

In summary, a memristor device with well unipolar resistive switching performances has been fabricated, for the first time, based on the single ZnO microwire and Ag electrodes. The single ZnO microwire memory is stable, rewritable, and nonvolatile with an on/off ratio over 1 × 10^3^, operating voltages less than 1 V, and high-endurance stability. Abnormally, the reset voltages are observed to be larger than the set voltages. The resistive switching could be explained by conducting filamentary mechanism. The conduction mechanisms dominating the low- and high- resistance states are proposed to be ohmic behavior and space-charge-limited current, respectively. The simple structure, large on/off ratio, and bistable performance of the device make it very attractive for nonvolatile resistive switching memory applications.

## Competing interests

The authors declare that they have no competing interests.

## Authors’ contributions

YH fabricated and measured the memory devices and drafted the manuscript. YL and ZHS assisted in the data analysis. GLY and HBZ revised the manuscript critically and made some changes. All authors read and approved the final manuscript.
